# Elesclomol induces copper‐dependent ferroptosis in colorectal cancer cells via degradation of ATP7A

**DOI:** 10.1002/1878-0261.13079

**Published:** 2021-09-15

**Authors:** Wei Gao, Zhao Huang, Jiufei Duan, Edouard C. Nice, Jie Lin, Canhua Huang

**Affiliations:** ^1^ State Key Laboratory of Biotherapy and Cancer Center West China Hospital West China School of Basic Medical Sciences and Forensic Medicine Sichuan University Collaborative Innovation Center for Biotherapy Chengdu China; ^2^ Department of Biochemistry and Molecular Biology Monash University Clayton Australia; ^3^ Department of Medical Oncology The Second Affiliated Hospital of Kunming Medical University Kunming China

**Keywords:** ATP7A, colorectal cancer, copper, elesclomol, ferroptosis

## Abstract

Cancer cells reprogram their copper metabolism to adapt to adverse microenvironments, such as oxidative stress. The copper chelator elesclomol has been reported to have considerable anticancer efficacy, but the underlying mechanisms remain largely unknown. In this study, we found that elesclomol‐mediated copper overload inhibits colorectal cancer (CRC) both *in vitro* and *in vivo*. Elesclomol alone promotes the degradation of the copper transporter copper‐transporting ATPase 1 (ATP7A), which retards the proliferation of CRC cells. This property distinguishes it from several other copper chelators. Combinational treatment of elesclomol and copper leads to copper retention within mitochondria due to ATP7A loss, leading to reactive oxygen species accumulation, which in turn promotes the degradation of SLC7A11, thus further enhancing oxidative stress and consequent ferroptosis in CRC cells. This effect accounts for the robust antitumour activity of elesclomol against CRC, which can be reversed by the administration of antioxidants and ferroptosis inhibitors, as well as the overexpression of ATP7A. In summary, our findings indicate that elesclomol‐induced copper chelation inhibits CRC by targeting ATP7A and regulating ferroptosis.

## Graphical abstract

In this study, we describe the molecular mechanisms by which elesclomol‐mediated copper (Cu^2+^) overload inhibits colorectal cancer (CRC) both *in vitro* and *in vivo*. Elesclomol elevated Cu^2+^ levels in mitochondria and decreased the expression of the Cu^2+^ transporter ATP7A, leading to Cu^2+^ retention and the subsequent accumulation of reactive oxygen species. The process promoted the degradation of SLC7A11, which further enhanced oxidative stress and induced ferroptosis in CRC cells.

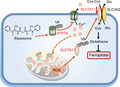

**Abbreviations**
ATP7A: copper‐transporting ATPase 1
Cav‐1: Caveolin‐1
CCS: copper chaperone for SOD1
CRC: colorectal cancer
FDX1: ferredoxin 1
GSH: reduced form of glutathione
IHC: immunohistochemistry
NAC: *N*‐acetyl‐l‐cysteine
ROS: reactive oxygen species
SOD: superoxide dismutase

## 1 Introduction

Metals are ubiquitous in the environment and play fundamental roles in every aspect of biological activity. Although very toxic species exist, several metals are indispensable for the proper function of biomacromolecules, such as calcium, magnesium, zinc, iron and copper [1]. Among them, copper has been reported to be an essential element in both physiological and pathological situations [2]. For instance, copper binds with tyrosinase, which is critical for the pigment formation [3]. Copper is also the ligand for the Cu/Zn superoxide dismutase (SOD1) which supports antioxidant function [4]. Dysregulation of copper metabolism, including excessive copper accumulation or improper transporting, has harmful effects [5]. This toxicity is partially attributed to improper binding with other protein sites, leading to misfolding, aggregation and loss of function of these proteins [6]. As a consequence, aberrant copper metabolism has been linked to many diseases, especially cancer [7, 8, 9].

Preferential copper trafficking primes cancer cells for their antioxidant defence in response to active metabolic patterns and the consequent accumulation of reactive oxygen species (ROS). Cancer cells upregulate several copper chaperones such as the copper chaperone for SOD1 (CCS), which binds cytosolic copper and transfers it to SOD1 [10]. Disruption of copper trafficking by pharmacologic inhibition of CCS has been shown to have therapeutic effects against a variety of cancers, indicating that proper trafficking is needed for cancer cell proliferation [11]. Similarly, another copper chaperone Atox1 has been shown to mediate metastasis of cancer cells by coordinating copper transport [12]. These data suggest that copper supports cancer initiation and progression in some cases. However, the concentration and transportation of intracellular copper must be precisely controlled due to its potential cytotoxicity. Excessive copper accumulation can disrupt mitochondrial respiration, which is a vulnerability of cancer cells with high basal ROS level. This property links copper to a form of ROS‐related cell death, namely, ferroptosis, which is characterized by elevated lipid peroxidation [13]. Importantly, cancer cells with therapeutic resistance frequently acquire sensitivity to ferroptosis. This fact provides opportunities for the treatment of tumour recurrence which is a current hotspot of cancer research activity [14, 15]. To date, extensive efforts have been taken to clarify ferroptosis as an iron‐dependent activity, but the role of copper metabolism in the regulation of ferroptosis is under‐explored, although emerging evidence has implicated its role in neurology [16, 17].

**Fig. 1:**
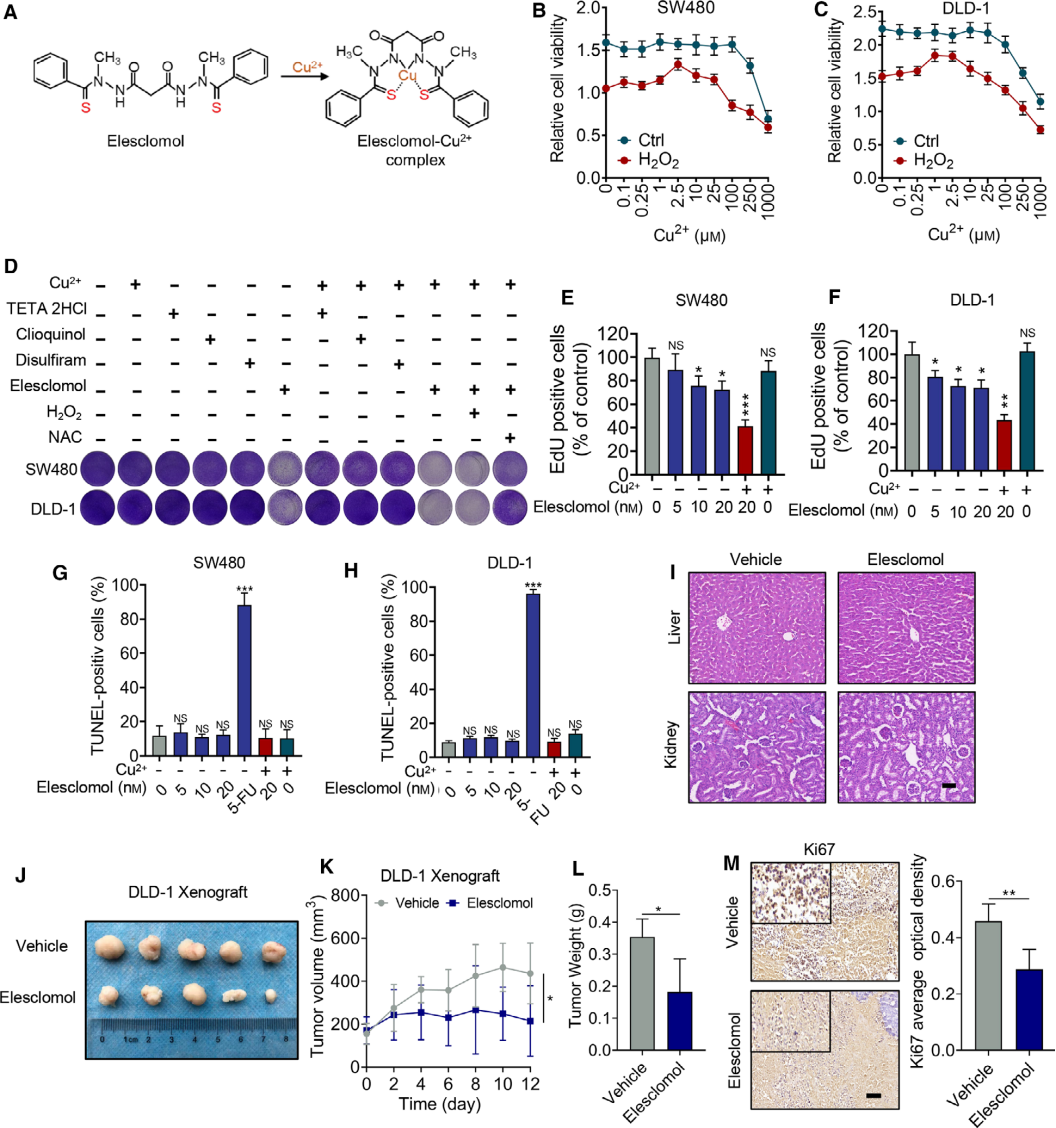
Elesclomol inhibits CRC growth both *in vitro* and *in vivo*. (A) Chemical structure of elesclomol before or after binding with copper. (B, C) Cells were treated with indicated concentration of copper (CuCl_2_) in the present or absence of H_2_O_2_ (100 μm) for 24 h followed by MTT assay to determine cell viability (*n* = 3). (D) Survival fraction assay showing the cell survival in the combinational treatment of indicated chelators (20 nm), CuCl_2_ (2 μm), H_2_O_2_ (100 μm) or NAC (1 μm) for 24 h (*n* = 3). (E, F) EdU assay showing EdU incorporation of cells on treatment with the indicated concentrations of elesclomol and 2 μm CuCl_2_ (*n* = 3). (G, H) TUNEL assay showing apoptosis of cells in the treatment as (E) and (F). 5‐FU was used as the positive control (*n* = 3). (I) H&E staining showing histomorphology of mice livers and kidneys (*n* = 5). Scale bars, 50 μm. (J, L) DLD‐1 cells were subcutaneously injected into nude mice followed by intraperitoneal treatment with elesclomol. The volume and weight of tumours were determined (*n* = 5). (M) IHC assay showing Ki67 staining of tumour xenografts in elesclomol‐treated and control group (*n* = 5). Scale bar, 50 μm. Data are means ± SEM from at least three independent repeats. The *P*‐value in (K) was determined by two‐way ANOVA, and the others were two‐tailed *t*‐test. *P < 0.05, **P < 0.01; ***P < 0.001.

Because of the vital role of copper metabolism in tumorigenesis, a variety of copper chelators have been considered for cancer treatment, such as elesclomol (also known as STA‐4783; Fig. 1A). Electrochemical studies showed that Cu^2+^ is coordinated with the N (nitrogen) and S (sulphur) atoms of elesclomol [18]. Then, copper‐binding elesclomol rapidly transports Cu^2+^ to mitochondria, where Cu^2+^ is reduced to Cu^+^, and ROS is simultaneously generated [19]. It has been shown that the copper‐transporting efficacy of elesclomol is superior to many other copper chelators [19]. This high efficacy might explain the significant anticancer activity observed in a board range of tumour types, including lung cancer, melanoma and sarcoma [20]. In a breast cancer study, elesclomol was reported to induce apoptosis [21]. However, the mechanism underlying its anticancer activity is not fully understood. Most recently, elesclomol was used to treat Menkes disease, a neurological disorder caused by copper deficiency [22]. These reports suggest that due to a distinct copper metabolism pattern between cancerous and noncancerous cells, elesclomol has potential to be a multifunctional drug candidate that kills cancer cells and protecting neural cells.

Here, we report that elesclomol‐mediated copper overload suppresses colorectal cancer (CRC) both *in vitro* and *in vivo*. We found that elesclomol, in contrast to several other copper chelators, downregulates the expression of the copper transporter copper‐transporting ATPase 1 (ATP7A) thus retarding the growth of CRC cells. This function is independent of copper chelation. When copper supplementation is provided, elesclomol binds extracellular and cytoplastic copper and brings them to mitochondria, leading to severe death of CRC cells through ROS accumulation and consequent ferroptosis, which requires the loss of ATP7A. These findings address the preference of copper metabolism in CRC and suggest the potential use of elesclomol in CRC treatment.

## 2 Materials and methods

### 2.1 Cell culture

Human cancer cell lines were purchased from the American Type Culture Collection (ATCC, Manassas, VA, USA). MIA‐PaCa‐2, Panc‐1, HeLa and C33A were cultured in RPMI‐1640 (Gibco, Manassas, VA, USA), and Huh7, PLC, DLD‐1 and SW480 were cultured in Dulbecco's modified Eagle medium (Gibco). Both media were supplemented with 100 U·mL^−1^ penicillin and streptomycin (Sigma, Kenilworth, NJ, USA) as well as 10% FBS (Biowest, Paris, France) at 37 °C in a 5% CO_2_ atmosphere.

### 2.2 Reagents and antibodies

Copper chelators elesclomol (S1052), clioquinol (S4601), TETA 2HCl (S6585) and disulfiram (S1680) were purchased from Selleck (Houston, TX, USA). Lipofectamine 3000 (L3000015) was obtained from Thermo Fisher Scientific (Manassas, VA, USA). Antibodies for β‐actin (#3700), HA‐Tag (#2367), Flag‐Tag (#14793), SLC7A11 (#12691), CD44 (#37259), Ubiquitin (#3936), OTUB1 (#3783) and Ki67 (#9449) were obtained from Cell Signaling Technology. Antibodies for ATP7A (ab131400) and ATP7B (ab131208) were purchased from Abcam (Cambridge, UK).

### 2.3 Animal model

Female nude mice (about 6 weeks old) were obtained from HFK Bioscience Co., Ltd (Beijing, China). Mice were maintained in the SPF Laboratory Animal Center of SKLB, West China Hospital, Sichuan University with humane care. The room temperature is kept as 22 °C, and the relative humidity is 55%. To establish the subcutaneous model, 1 × 10^7^ DLD‐1 cells were suspended in PBS and injected into mice. Seven days postinjection, and mice were grouped randomly and received elesclomol (80 mg·kg^−1^·day^−1^) or PBS treatment for another 12 days. Tumour volume was measured every two days. Mice were then euthanized, and tumours fixed in formalin prior to immunohistochemistry (IHC) staining. Animal study was approved by the Institutional Animal Care and Treatment Committee of Sichuan University.

### 2.4 Immunoblotting and immunoprecipitation

For Western blotting, cells were harvested and washed in cold PBS twice before lysis using RIPA buffer (1% Triton X‐100, 1% deoxycholate, 0.1% SDS supplemented with protease inhibitor and phosphatase inhibitor) with sonication. Protein lysates were then separated on SDS/PAGE and transferred to a PVDF membrane (EMD Millipore, ISEQ00010, Kenilworth, NJ, USA), which was blocked by 5% FBS for 1 h at room temperature. Next, the membrane was cut into strips according to the molecular weight of the targeted proteins and incubated with primary antibodies at 4 °C with gentle shaking overnight. Strips were then washed in TBST, followed by incubation with secondary antibodies for 1 h at room temperature. Next, strips were washed in TBST and incubated with Immobilon Western HRP Substrate (EMD Millipore, WBKLS0500), and images were captured using a ChemiScope 6000 Touch (Clinx, Shanghai, China).

For immunoprecipitation, cells were lysed using IP lysis buffer (100 mm NaCl, 0.5 mm EDTA, 20 mm Tris/HCl, 0.5% NP‐40). Then, cell lysate was incubated with 1 μg primary antibody at 4 °C with gently shaking overnight. Next, protein A agarose beads (GE Healthcare, 17‐0963‐03, Chicago, IL, USA) were added to the samples for 2 h at 4 °C with gently shaking before washing with IP wash buffer (150 mm NaCl, 0.5 mm EDTA, 20 mm Tris/HCl, 0.5% NP‐40) and separation by SDS/PAGE as described above.

### 2.5 Determination of cell viability, proliferation and cell death

For MTT assay, cells were seeded at 3000 cells per well in 96‐well plates. When cells were adherent and had morphologically spread, cells were treated with indicated drugs for 24 h. MTT (5 mg·mL^−1^; Sigma, M2128) was added followed by culture for 3 h. The absorbance was determined at 570 nm using a spectrophotometer.

For the colony formation assay, cells were seeded at 300 cells per well in 24‐well plates and treated for 15 days. Then, cells were washed with cold PBS twice, fixed in 4% paraformaldehyde and stained using crystal violet. Images were captured using a Molecular Imager Gel Do XR+ System (BIO‐RAD, Hercules, CA, USA), and clone numbers were calculated according to manual instructions.

For the cell survival fraction assay, cells were seeded at 50 000 cells per well in 24‐well plates and treated for 24 h. Then, samples were handled as for the colony formation assay except clone numbers were counted.

For EdU assay, cells were seeded at 3000 cells per well in 96‐well plates and treated for 24 h. Then, cells were labelled with 50 μm EdU (RiboBio, Guangzhou, China) for 2 h followed by fixation using 4% paraformaldehyde. Images were captured by fluorescence microscopy (ZEISS, Oberkochen, Germany), and quantification was performed according to the manufacturer’s instructions.

For TUNEL assay, cells were seeded at 3000 cells per well on glass coverslips in 24‐well plates and treated for 24 h. Then, cells were fixed using 4% paraformaldehyde and assays performed according to the manufacturer’s instructions (Promega, Chicago, IL, USA). Images were captured as for the EdU assay.

### 2.6 RNA interference and quantification

For RNA interference, siRNA was purchased from GenePharma (Shanghai, China). The sequence of siATP7A is as follows: 5′‐UAUCCUAUGGUUAAACCUCUG‐3′. The siRNA was transfected into indicated cells using Lipofectamine 3000 for 48 h according to the manufacturer’s instructions, followed by qPCR to verify the efficacy of interference.

For quantitative RT‐PCR assay, total RNA was obtained using TRIzol (Thermo Fisher Scientific, 15596018) and reverse transcribed using a RT kit (Takara, RR047A, Mountain View, CA, USA). The mRNA level was determined using SYBR Green reagent (Bio‐Rad, 1725271). The sequences of primers are listed in the Table S1.

### 2.7 Immunohistochemistry

Fixed tumour xenografts were embedded in paraffin for sectioning. Sections were rehydrated before antigen retrieval using citrate buffer. Then, sections were blocked by FBS for 1 h at room temperature, followed by incubation with primary antibodies at 4 °C overnight. Next, sections were washed in PBS and incubated with secondary antibodies for 30 min at room temperature. Then, sections were washed in PBS and stained by DAB reagents for microscopy.

### 2.8 Statistical analysis

Data were processed using graphpad prism 8 software (GraphPad Software, San Diego, CA, USA). *P*‐value was determined using two‐tailed Student's *t*‐test, two‐way ANOVA or log‐rank (Mantel–Cox) test. Data are shown as means ± SEM. At least three independent experiments were repeated. *P*‐value lower than 0.05 is considered as statistically significant. *P < 0.05, **P < 0.01; ***P < 0.001.

## 3 Results

### 3.1 Elesclomol inhibits colorectal cancer growth both *in vitro* and *in vivo*

To determine the antitumour activity of elesclomol, cell viability assays were performed on a panel of cancer cell lines including pancreatic cancer (MIA‐PaCa‐2, Panc‐1), cervical carcinoma (HeLa, C33A), liver cancer (Huh7, PLC/PRF/5) and CRC (SW480, DLD‐1). Among them, elesclomol inhibited the growth of CRC cells with the efficacy. This high cytotoxicity was further confirmed by the colony formation assay (Fig. S1A–C). Additionally, the anti‐CRC activity of elesclomol overrides several other copper chelators including clioquinol, TETA 2HCl and disulfiram (Fig. S1D,E). Given that the gastrointestinal tract is the main organ to absorb copper in the human body, the anti‐CRC effect of elesclomol might be attributed to dysregulated copper metabolism. To determine the importance of copper for CRC cells, we investigated the impact of exogenous copper on cell viability in the challenge of hydrogen peroxide‐induced oxidative stress. As shown in Fig. 1B,C, at a particular concentration (about 1–3 μm), copper supported the growth of CRC cells under oxidative stress, whereas high levels of copper (above 100 μm) are cytotoxic. This cytoprotective effect of copper might be related to the antioxidant function of SOD1, which requires copper as the ligand.

To ascertain if this anti‐CRC activity of elesclomol is caused by oxidative stress, a survival fraction assay was performed using the antioxidant *N*‐acetyl‐l‐cysteine (NAC). In the presence of exogenous copper, elesclomol, but not other copper chelators, induced significant cell death in CRC cells, which can be partially restored by the administration of NAC (Fig. 1D and Fig. S1F,G). This finding suggests that elesclomol‐induced CRC cell death is dependent of ROS accumulation in the combination of copper supplementation. Interestingly, in the absence of exogenous copper the proliferation of CRC cells was also retarded by elesclomol, though this effect was less potent than in combination with exogenous copper (Fig. 1D and Fig. S1F,G). Similarly, EdU assay also demonstrated significant suppression of CRC growth by elesclomol (Fig. 1E,F and Fig. S1H). However, no obvious DNA damage or cleaved caspase‐3 was detected suggesting that elesclomol‐induced CRC cell death may not be caused by apoptosis (Fig. 1G,H and Fig. S1I,J), which is somewhat contradictory to previously published results [21]. This difference might be due to distinct copper metabolism in different cancer types.

To determine the anti‐CRC effect of elesclomol *in vivo*, we generated a subcutaneous xenograft mouse model with or without elesclomol treatment. As shown in Fig. 1I, treatment of elesclomol has no obvious toxicity to several other normal organs including liver and kidney, suggesting an acceptable safety profile for this drug. In contrast, elesclomol was shown to suppress tumour growth as evidenced by the reduced weight and volume of xenografts (Fig. 1J,L). Furthermore, IHC assay showed weaker expression of Ki67 in tumours of the elesclomol‐treated group (Fig. 1M). Taken together, these evidences indicate that elesclomol inhibits the growth of CRC both *in vitro* and *in vivo*.

### 3.2 Combinational treatment of elesclomol and copper induces ferroptosis

**Fig. 2:**
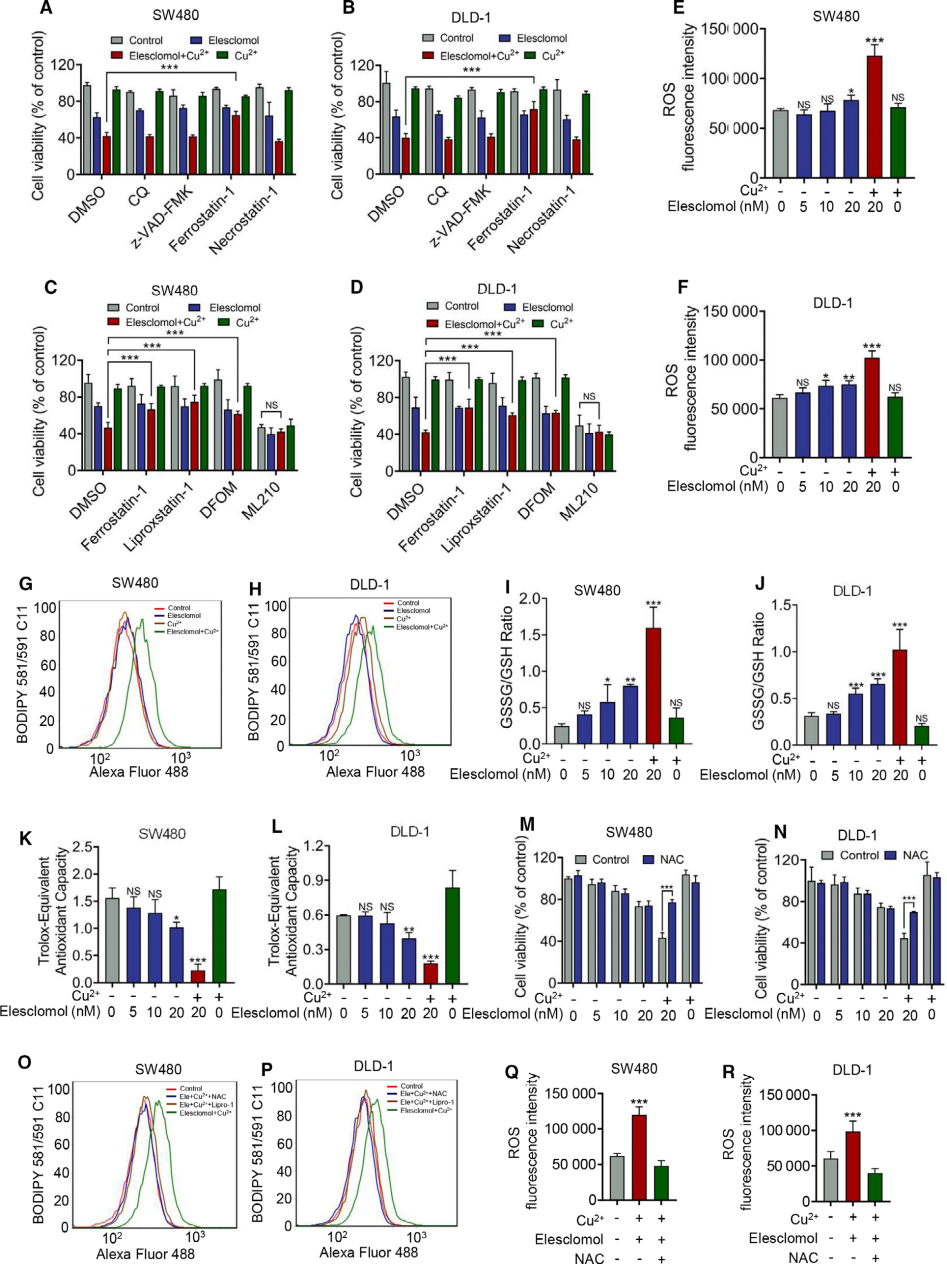
Combinational treatment of elesclomol and copper induces ferroptosis. (A, B) MTT assay showing cell viability of cells treated with indicated death inhibitors, CuCl_2_ (2 μm) and elesclomol (20 nm) for 24 h (*n* = 3). The concentration of inhibitors are as follows: CQ (10 μm), z‐VAD‐FMK (20 μm), Ferrostatin‐1 (4 μm) and Necrostatin‐1 (10 μm). (C, D) Cell viability was determined by MTT assay as (A) and (B; *n* = 3). The concentration of Liproxstatin‐1 and DFOM is 4 μm and 5 μm, respectively. ML210 (1 μm) was used as a positive control. (E, F) Cells were treated with indicated concentration of elesclomol with or without the combination of 2 μm CuCl_2_ for 24 h. ROS level was determined from fluorescence (*n* = 3). (G, H) Cells were treated with 2 μm CuCl_2_ or 20 nm elesclomol solely or in combination for 24 h, followed by detection of lipid ROS using BODIPY 581/591 C11 reagent (Invitrogen D3861, Carlsbad, CA, USA) using flow cytometry (*n* = 3). (I, J) Cells were treated as (E) and (F) followed by measurement of GSSG and GSH level using GSSG/GSH Assay Kit (Beyotime S0053, Shanghai, China; *n* = 3). (K, L) Cells were treated as (E) and (F), and their antioxidant capacity was determined by T‐AOC Assay Kit (Beyotime S0119) (*n* = 3). (M, N) Cells were treated as (E) and (F) with or without the supplementation of 1 mm NAC, followed by detection of cell viability by MTT assay (*n* = 3). (O, P) Cells were treated, and lipid ROS was detected as (G) and (H) with or without the administration of 1 mm NAC or 4 μm liproxstatin‐1 (*n* = 3). (Q, R) In the presence of 20 nm elesclomol and 2 μm CuCl_2_, cells were treated with or without 1 mm NAC followed by detection of ROS level (*n* = 3). Data are mean ± SEM from at least three independent repeats. The *P*‐values were determined by two‐tailed *t*‐test. *P < 0.05, **P < 0.01; ***P < 0.001.

To determine which form of death is responsible for the antitumour activity of elesclomol, inhibitors for different types of cell death including autophagy (CQ), apoptosis (z‐VAD‐FMK), ferroptosis (Ferrostatin‐1) and necrosis (Necrostatin‐1) were applied in an attempt to restore the viability of CRC cells treated by elesclomol and copper. The results showed that ferrostatin‐1, but not other inhibitors, significantly restored cell viability (Fig. 2A,B). To further confirm this, several other inhibitors of ferroptosis including liproxstatin and DFOM were also shown to restore cell viability, and a ferroptosis inducer, ML210, repressed CRC cells to a comparable level to the elesclomol and copper combinational treated group (Fig. 2C,D). These observations suggest that ferroptosis is probably the major cell death form induced by combinational treatment of elesclomol and copper. As ferroptosis is characterized by ROS accumulation, ROS levels within CRC cells were measured. As shown in Fig. 2E,F, treatment of elesclomol alone induces a slight elevation of ROS, in contrast to a robust oxidative stress by combinational treatment of elesclomol and copper. Consistently, combinational but not single treatment with elesclomol elevated lipid ROS in CRC cells (Fig. 2G,H). In addition, combinational treatment of elesclomol and copper consumed a large amount of the reduced form of glutathione (GSH) and suppressed the antioxidant capacity of CRC cells (Fig. 2I–L).

To ascertain if ROS accumulation contributes to ferroptosis induced by combinational treatment of elesclomol and copper, NAC was used as an exogenous antioxidant. It was found that NAC partially restored the activity of CRC cells in the combinational, but not the sole group (Fig. 2M,N). Furthermore, treatment of NAC was shown to suppress the accumulation of lipid ROS (Fig. 2O,P). To validate the antioxidant efficacy of NAC, we also measured the ROS level in the presence of NAC in cells treated with elesclomol and Cu^2+^. As shown in Fig. 2Q,R, treatment of NAC significantly decreases the ROS level in indicated cells. Taken together, these results indicate that elesclomol solely inhibits the proliferation of CRC cells with a considerable effect independent of ferroptosis, whereas combinational treatment of elesclomol and copper dramatically elevates lipid ROS and induces robust ferroptosis.

### 3.3 Elesclomol induces intracellular copper retention via ATP7A degradation

**Fig. 3:**
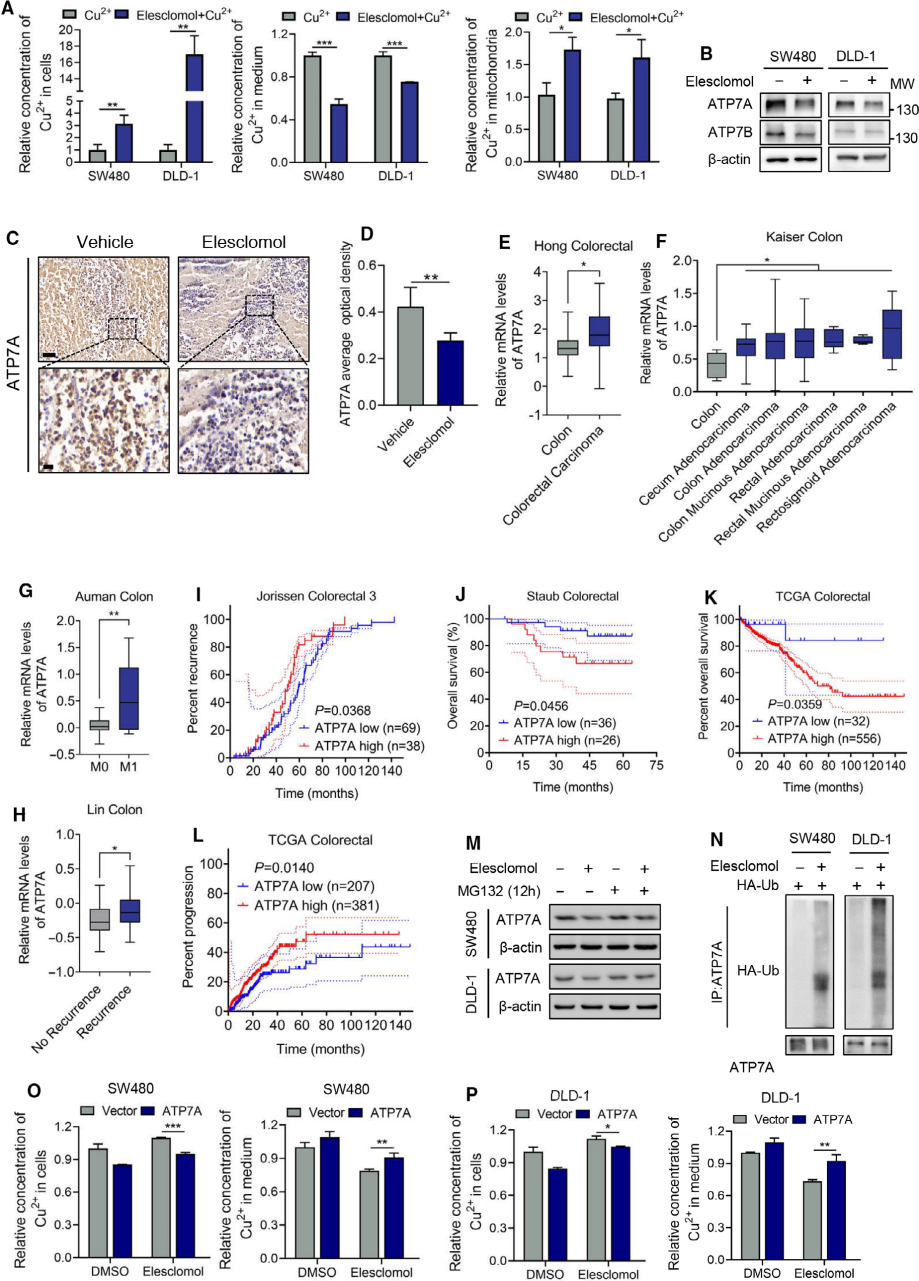
Elesclomol induces intracellular copper retention via ATP7A degradation. (A) Cu^2+^ levels in cytoplasm, mitochondria and medium were measured using Copper (Cu) Colorimetric Assay Kit (Elabscience, E‐BC‐K300‐M) after exogenous CuCl_2_ (2 μm) was added (*n* = 3). (B) Western blot showing the protein level of ATP7A and ATP7B following treatment with 20 nm elesclomol in the absence of exogenous CuCl_2_ (*n* = 3). (C, D) Tumour xenografts were stained with ATP7A by IHC assay (*n* = 5). Scale bar, 50 μm (top) or 20 μm (bottom). (E, F) Hong Colorectal and Kaiser Colon Datasets were explored for ATP7A expression in normal colon and CRC tissue. (G) The expression of ATP7A in nonmetastatic CRC tissue (M0) and metastatic CRC tissue (M1) according to Auman Colon Dataset. (H) Dataset Lin Colon was explored to determine the ATP7A level in CRC patients with or without recurrence at 5 years. (I–L) Kaplan–Meier analysis showing overall and progression‐free survival of CRC patients with high or low ATP7A expression. Patients were grouped by the auto‐selected best cut‐off. (M) Western blot showing the protein degradation of ATP7A by using proteasome inhibitor MG132 (20 μm) (*n* = 3). (N) HA‐Ub plasmid was transiently transfected into indicated cells followed by co‐IP assay to determine the ubiquitination of ATP7A (*n* = 3). (O, P) Exogenous ATP7A was overexpressed in indicated cells followed by measurement of Cu^2+^ level in cells and medium as (A) (*n* = 3). Data are means ± SEM from at least three independent repeats. The *P*‐values in (I–L) were determined by log‐rank (Mantel–Cox) test, and others were two‐tailed *t*‐test. *P < 0.05, **P < 0.01; ***P < 0.001

To investigate whether copper chelation by elesclomol contributes to its antitumour function, the concentration of copper in the cell fraction and medium was analysed. Combinational treatment of copper and elesclomol led to an increase of copper in cytoplasm and mitochondria, but a decrease in medium, suggesting that elesclomol brings copper from the medium into the mitochondria of cells (Fig. 3A). Generally, copper transportation is largely dependent on ATP7A and ATP7B, two transporters that are located at the cytoplasmic membrane and mediate copper efflux. Therefore, the expression of ATP7A and ATP7B without adding exogenous copper was determined. Elesclomol suppressed the level of ATP7A in both CRC cell lines, whereas ATP7B was downregulated in SW480 cells but failed to be repressed in DLD‐1 cells (Fig. 3B and Fig. S2A,B). Given the consistent phenotypes observed in both CRC cell types, we postulated that elesclomol‐induced downregulation of ATP7A, but not ATP7B, is responsible for copper retention and consequent antitumour activity of elesclomol. In addition, IHC staining in xenografts also showed a decrease of ATP7A expression in the elesclomol‐treated group, indicating that elesclomol downregulates ATP7A both *in vitro* and *in vivo* (Fig. 3C,D).

To determine the pathological significance of ATP7A in CRC, several datasets [23, 24, 25, 26, 27, 28, 29] were explored which showed that ATP7A is upregulated in CRC compared with normal tissue (Fig. 3E,F). Moreover, upregulation of ATP7A is associated with metastasis and recurrence of CRC (Fig. 3G,H). Consistently, overexpression of ATP7A is related to poor overall survival and progression‐free survival of CRC patients (Fig. 3I–L). These results indicate that CRC cells upregulate ATP7A to facilitate tumour growth and progression, further suggesting that elesclomol holds therapeutic potential.

To identify the mechanism underlying elesclomol‐induced ATP7A downregulation, the mRNA level of ATP7A following treatment with elesclomol was determined. As shown in Fig. S2C, the mRNA level was not obviously altered by elesclomol, suggesting that elesclomol‐mediated downregulation of ATP7A might be related to protein degradation rather than gene transcription. To confirm it, the proteasome inhibitor MG132 was used to block protein degradation. Elesclomol failed to induce a decrease on ATP7A protein levels, indicating that elesclomol downregulates ATP7A via a protein degradation pathway (Fig. 3M and Fig. S2D). Generally, protein degradation is mediated by ubiquitination, and therefore, a co‐immunoprecipitation assay was performed to demonstrate ATP7A ubiquitination induced by elesclomol (Fig. 3N). To further explored the mechanisms underlying elesclomol‐induced ATP7A degradation, we investigated potential key factors that have been reported to be involved in the degradation of ATP7A. Such factors include Caveolin‐1 (Cav‐1), which binds with ATP7A to protect ATP7A from proteasome‐mediated degradation in endothelial cells [30]. However, our results showed that the expression of Cav‐1 was not obviously changed upon treatment of elesclomol, suggesting that Cav‐1 may not be involved in elesclomol‐induced ATP7A degradation in CRC cells (Fig. S2E). Moreover, another copper transporter CTR1, which is responsible for copper uptake, remains unchanged upon elesclomol treatment (Fig. S2E). To determine whether loss of ATP7A is required for elesclomol‐induced copper retention in CRC cells, exogenous ATP7A was overexpressed in CRC cells. ATP7A overexpression was seen to abolish the copper efflux caused by elesclomol treatment, indicating that elesclomol induces copper retention within CRC cells via ATP7A degradation (Fig. 3O,P).

### 3.4 Elesclomol‐mediated ROS accumulation involves the degradation of SLC7A11

**Fig. 4:**
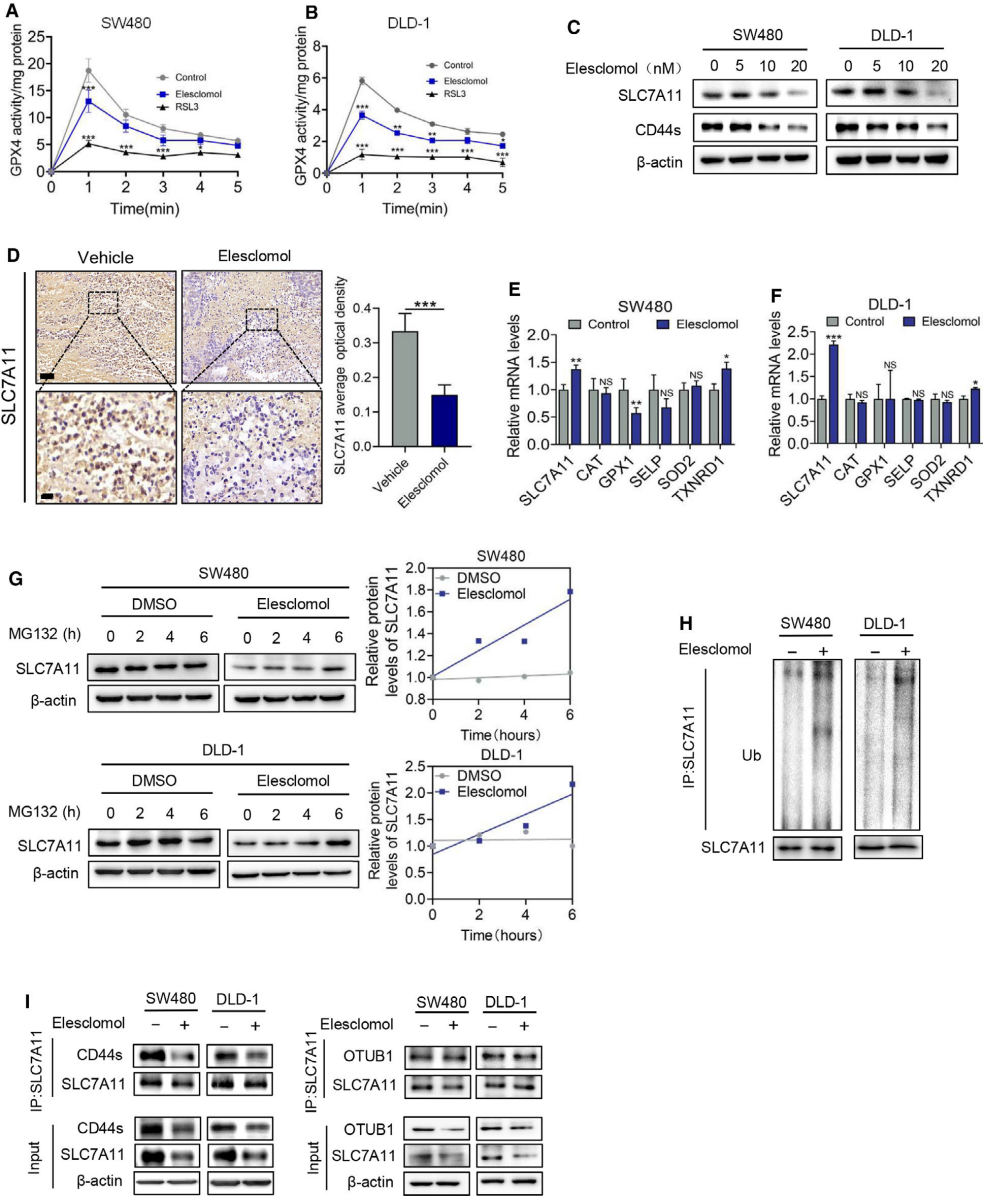
Elesclomol‐mediated ROS accumulation involves the degradation of SLC7A11. (A, B) GPX4 activity was determined using GPX4 Kit (Beyotime S0056) following treatment with elesclomol (20 nm) for indicated time (*n* = 3). The GPX4 inhibitor RSL3 (2 μm) was used as a positive control. (C) Western blot showing the protein level of SLC7A11 and CD44 with elesclomol treatment at indicated concentration for 24 h (*n* = 3). (D) IHC assay showing the expression of SLC7A11 in tumour xenograft (*n* = 5). Scale bar, 50 μm (top) or 20 μm (bottom). (E, F) qPCR showing the mRNA level of indicated antioxidant genes on treatment with elesclomol (20 nm) for 24 h (*n* = 3). (G) Cells were treated with 20 nm elesclomol and 20 μm MG132 for indicated time followed by detection of SLC7A11 expression by immunoblotting (*n* = 3). (H) The ubiquitination of SLC7A11 was measured following treatment with 20 nm elesclomol for 24 h via co‐IP assay (*n* = 3). (I) co‐IP assay showing the interaction of SLC7A11 with CD44 and OTUB1 following treatment with 20 nm elesclomol for 24 h (*n* = 3). Data are means ± SEM from at least three independent repeats. The *P*‐values were determined by two‐tailed *t*‐test. *P < 0.05, **P < 0.01; ***P < 0.001.

Ferroptosis is frequently induced by loss of SLC7A11, a transporter responsible for the production of GSH. Deficient GSH production leads to a decrease in GPX4 activity, resulting in lipid peroxidation and ferroptosis. To determine the mechanism underlying elesclomol‐induced ferroptosis, GPX4 activity and SLC7A11 expression were measured following treatment with elesclomol. As shown in Fig. 4A,B, elesclomol induced a decrease of GPX4 activity, through the efficacy is less potent than GPX4 inhibitor RSL3. Elesclomol also downregulated the protein levels of SLC7A11 and its binding partner CD44 (Fig. 4C,D and Fig. S3A,B). These results are consistent with our previous observations that elesclomol suppressed the GSH level in CRC cells (Fig. 2I,J). Surprisingly, the mRNA level of SLC7A11 is not decreased, but even increased by treatment with elesclomol, suggesting that elesclomol regulates protein stability, but not the transcription of SLC7A11 (Fig. 4E,F). This hypothesis was confirmed by the findings that elesclomol promoted the ubiquitination and subsequent degradation of SLC7A11 (Fig. 4G,H). CD44 and OTUB1 have been reported to bind with SLC7A11 thus regulating its protein stability. We therefore determined their interaction during treatment with elesclomol. As shown in Fig. 4I, treatment with elesclomol repressed the interaction of SLC77A11 with CD44, but not with OTUB1. Taken together, these results suggest that elesclomol promotes degradation of SLC7A11, which may lead to ferroptosis.

To determine the pathological significance of SLC7A11 in CRC, we explored several datasets [23, 24, 31, 32, 33, 34, 35, 36]. It was found that SLC7A11 is overexpressed in cancerous tissue compared with normal tissue, and the high expression of SLC7A11 is also correlated with CRC metastatic recurrence (Fig. S3C–J). These data indicate that CRC cells upregulate SLC7A11 to support cancer growth under oxidative stress, suggesting a rationale for elesclomol‐induced SLC7A11 downregulation for the treatment of CRC.

### 3.5 Elesclomol‐induced loss of ATP7A is required for SLC7A11 degradation

**Fig. 5:**
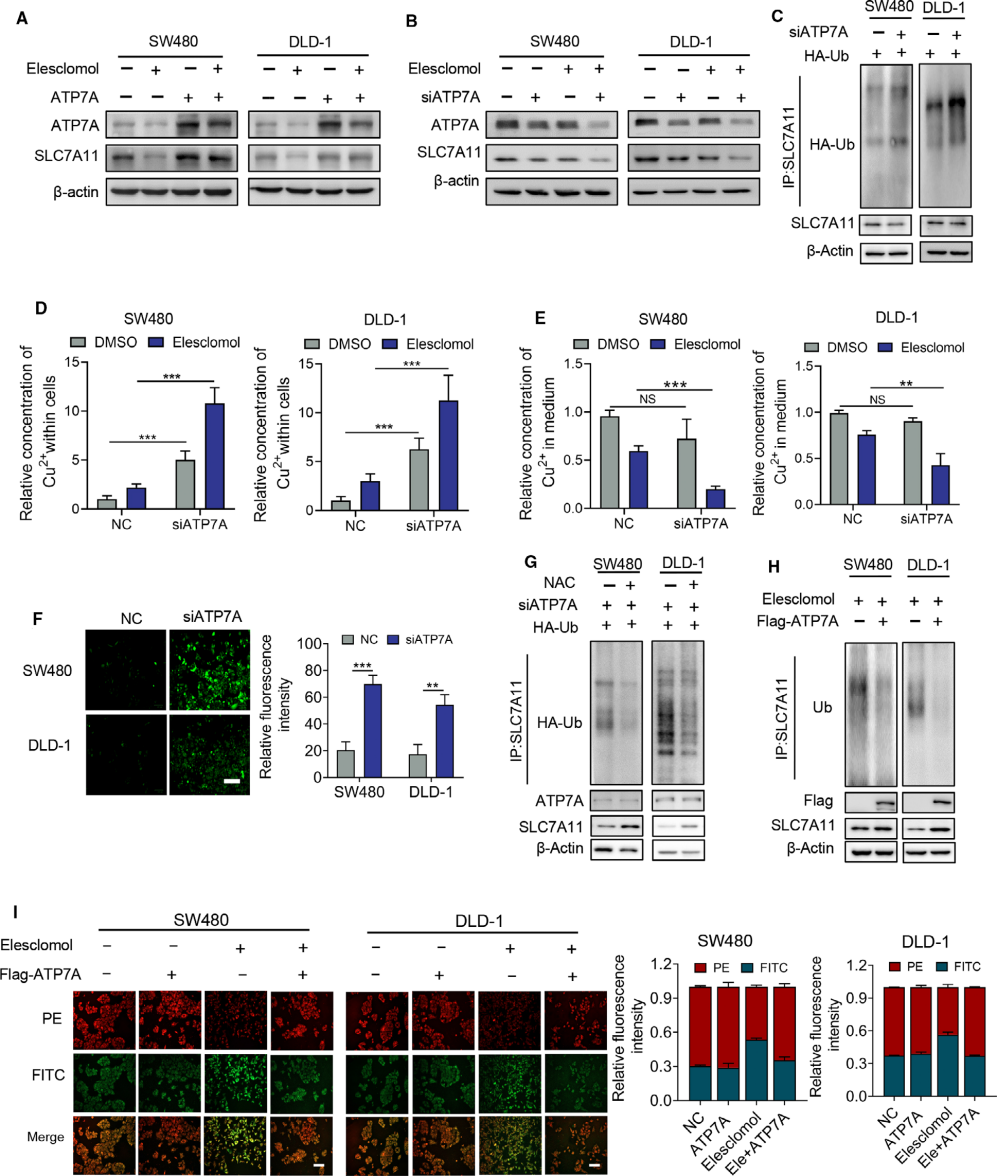
Elesclomol‐induced loss of ATP7A is required for SLC7A11 degradation. (A) Exogenous ATP7A was overexpressed in cells treated with 20 nm elesclomol for 24 h followed by Western blot to show the protein expression of SLC7A11 (*n* = 3). (B) Endogenous ATP7A was knocked down by siRNA and then treated as (A) (*n* = 3). (C) Endogenous ATP7A was knocked down in indicated cells followed by co‐IP assay to determine the ubiquitination of SLC7A11 (*n* = 3). (D, E) Levels of Cu^2+^ in cells and medium were measured after the knockdown of ATP7A (*n* = 3). (F) Immunofluorescence assay showing the ROS level in cells with or without knockdown of ATP7A (*n* = 3). Scale bar, 50 μm. (G) Cells in (C) were treated with or without antioxidant NAC (1 mm) for 24 h followed by detection of the ubiquitination of SLC7A11 (*n* = 3). (H) Exogenous ATP7A was overexpressed in cells treated with 20 nm elesclomol for 24 h followed by co‐IP assay to determine the ubiquitination of SLC7A11 (*n* = 3). (I) Immunofluorescence assay showing lipid ROS level in cells with or without overexpression of exogenous ATP7A (*n* = 3). PE (red) and FITC (green) represent reduced and oxidized form of probes, respectively. Scale bar, 50 μm. Data are means ± SEM from at least three independent repeats. The *P*‐values were determined by two‐tailed *t*‐test. **P < 0.01; ***P < 0.001.

We previously showed that elesclomol induces the degradation of both ATP7A and SLC7A11 (Figs 3 and 4), but the causal relationship between them is not clear. To clarify this, exogenous ATP7A was overexpressed in CRC cells. Restoration of SLC7A11 expression was observed in the elesclomol‐treated group (Fig. 5A and Fig. S4A,B). Additionally, knockdown of endogenous ATP7A using siRNA mimics the effect of elesclomol in decreasing SLC7A11, indicating that elesclomol‐induced SLC7A11 downregulation is dependent on ATP7A degradation (Fig. 5B and Fig. S4C–E). Moreover, knockdown of endogenous ATP7A is sufficient to induce ubiquitination of SLC7A11 and copper retention in cells (Fig. 5C–E).

To investigate the regulation of oxidative stress by ATP7A, ROS levels in cells after ATP7A knockdown were determined. As shown in Fig. 5F, knockdown of ATP7A led to an increase in ROS levels. Importantly, administration of NAC abolished the ubiquitination of SLC7A11 induced by ATP7A deficiency, indicating that loss of ATP7A leads to ROS accumulation, thereby resulting in SLC7A11 degradation thus further enhancing oxidative stress (Fig. 5G). Consistently, overexpression of ATP7A decreased the ubiquitination of SLC7A11 and ROS accumulation induced by elesclomol (Fig. 5H,I). These data suggest that elesclomol downregulates ATP7A to block copper efflux, leading to copper retention within cells and subsequent ROS production. ROS‐mediated SLC7A11 degradation then further enhances oxidative stress induced by elesclomol.

### 3.6 Elesclomol inhibits colorectal cancer through degradation of ATP7A

**Fig. 6:**
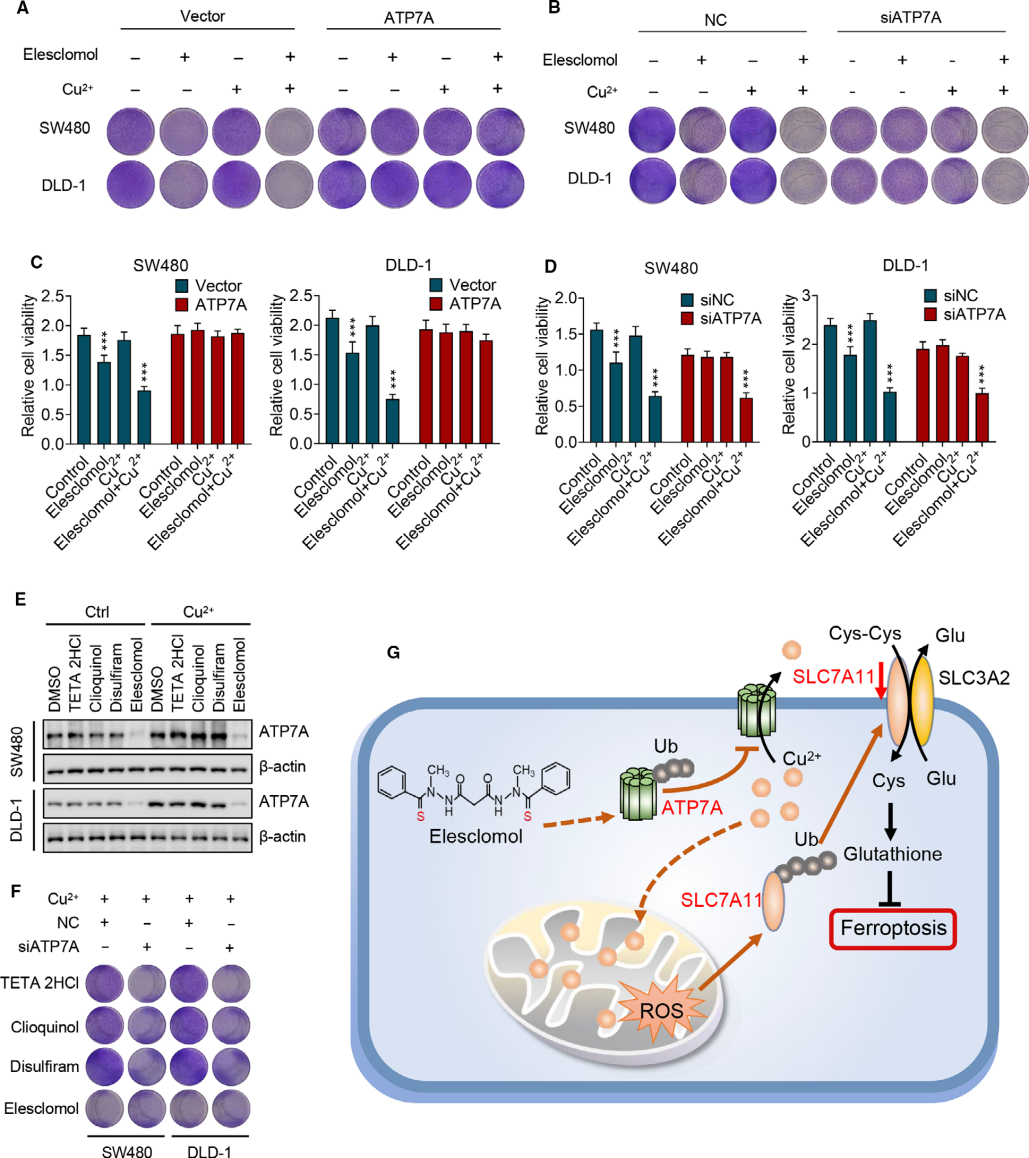
Elesclomol inhibits CRC through degradation of ATP7A. (A) Survival fraction assay showing the cell survival on treatment with elesclomol (20 nm) and Cu^2+^ (2 μm) for 24 h with or without the overexpression of exogenous ATP7A (*n* = 3). (B) Cells were treated as (A) with or without the knockdown of endogenous ATP7A (*n* = 3). (C, D) MTT assay showing the cell viability of cells treated as (A) and (B) (*n* = 3). (E) Cells were treated with indicated copper chelators (20 nm) for 24 h with or without the supplementation of 2 μm Cu^2+^ followed by immunoblotting to determine the expression of ATP7A (*n* = 3). (F) Survival fraction assay showing the cell survival in the treatment of indicated copper chelators (20 nm) in combination with 2 μm Cu^2+^ or ATP7A silencing (*n* = 3). (G) Working model of elesclomol‐induced ATP7A degradation and CRC ferroptosis. Elesclomol elevates the Cu^2+^ level in mitochondria and decreases the expression of ATP7A, leading to Cu^2+^ retention within cells and consequent ROS accumulation. This effect promotes the degradation of SLC7A11, which further enhances oxidative stress, eventually leading to ferroptosis in CRC cells. Data are means ± SEM from at least three independent repeats. The *P*‐values were determined by two‐tailed *t*‐test. ***P < 0.001.

To determine whether ATP7A degradation contributes to the antitumour activity of elesclomol, exogenous ATP7A was overexpressed during the combinational treatment with elesclomol and copper, followed by a survival fraction assay to detect proliferation. Overexpression of ATP7A restored the proliferation of CRC cells (Fig. 6A and Fig. S5A). By contrast, knockdown of ATP7A mimics the inhibitory effect of elesclomol (Fig. 6B and Fig. S5B). Consistently, MTT assay showed similar results (Fig. 6C,D). These data indicate that elesclomol inhibits CRC through degradation of ATP7A.

The effects of other copper chelators to regulate the expression of ATP7A were then investigated. As shown in Fig. 6E and Fig. S5C, ATP7A was upregulated in response to exogenous copper, which is exclusively abrogated by treatment with elesclomol, but not other copper chelators. This observation suggests that the reason why elesclomol shows stronger anti‐CRC activity compared with other chelators might be due to its ability to induce ATP7A degradation. To verity this, CRC cell proliferation was determined when ATP7A was knocked down followed by treatment with other chelators. As shown in Fig. 6F and Fig. S5D, knockdown of ATP7A enhanced the antitumour activity of other chelators to a comparable level to elesclomol. Taken together, these results indicate that elesclomol promotes degradation of ATP7A, leading to copper retention in cells, which results in ROS accumulation and ferroptosis of CRC cells, thus exhibiting antitumour activity (Fig. 6G).

## 4 Discussion

Here, we show that elesclomol‐induced copper overload inhibits CRC with high efficiency, which overrides several other copper chelators. This unique feature might be attributed to its ability to degrade ATP7A, a critical transporter responsible for copper efflux. In the presence of copper supplementation, elesclomol‐mediated copper retention in mitochondria leads to ROS accumulation, which further results in SLC7A11 downregulation and consequent ferroptosis of CRC cells. This mechanism contributes to the robust anti‐CRC activity of elesclomol. Interestingly, we also found that in the absence of copper supplementation, elesclomol‐mediated ATP7A degradation retards the proliferation of CRC cells without inducing obvious ferroptosis. This observation suggests additional functions of ATP7A apart from mediating copper transport.

Elesclomol was originally used as an apoptosis stimulator in the treatment of nonsmall cell lung cancer, melanoma and sarcoma [37]. The antitumour activity of elesclomol was reported to derive from the disruption of the cytoskeleton in tumour cells [37]. This mechanism leads to a combinational therapeutic strategy with paclitaxel, another drug inducing cytoskeleton disruption to enhance cytotoxicity in tumour cells [20]. In a phase II clinical trial, elesclomol plus paclitaxel resulted in a prolonged progression‐free survival of melanoma patients [38]. Combinational treatment of elesclomol and paclitaxel in a phase I clinical trial in cancer patients has been shown to be as well tolerated as treatment with paclitaxel alone [20]. The above evidence indicates that elesclomol is potentially a cancer therapeutic agent with encouraging efficacy and acceptable safety. In mechanistic studies, elesclomol has been reported to induce oxidative stress in melanoma and leukaemia cells, leading to cell apoptosis, which can be abrogated by treatment with antioxidant NAC [39]. Similarly, elesclomol has been also shown to suppress the growth of cisplatin‐resistant lung cancer cells by elevating ROS [40]. The capability of elesclomol to stimulate ROS is largely due to targeting mitochondrial electron transport [41]. In this study, we found that elesclomol‐mediated ROS accumulation induces ferroptosis, but not the widely reported apoptosis, in CRC cells. This observation implies that elesclomol kills cancer cells with different cell death mechanisms depending on the particular tumour type. Given that the intestine is the major organ responsible for copper absorption, CRC cells might be highly susceptible and shown a different cell death pattern in response to elesclomol treatment.

Ferroptosis is generally regarded as an iron‐related cell death pattern, while the role of other metals, such as copper, in this process has been largely ignored. Recently, copper and nickel have been shown to protect neuronal cells from ferroptosis thus ameliorating neurodegeneration and indicating that ferroptosis can be regulated by alternative metals to iron [16]. There are at least two possible reasons why copper can affect ferroptosis. Firstly, there is metabolic crosstalk between copper and iron, and thus, copper regulates ferroptosis through copper–iron interactions [42]. It has been shown that copper deficiency results in iron deficiency in rats during perinatal development [43]. Mechanistically, copper deficiency reduces iron absorption, leading to decreased loading of iron into transferrin, which eventually blocks the delivery of iron into erythroid cells, resulting in anaemia [44]. Secondly, copper is linked to ferroptosis by regulating ROS accumulation in cells, and this mechanism may be independent of iron metabolism. Copper has the ability to catalyse redox reactions and mimic pro‐oxidative features in zebrafish and mammal models [45, 46]. Given that iron‐mediated ferroptosis is largely attributed to lipid hydroperoxide induced by the Fenton reaction, copper is probably sufficient for the stimulation of ferroptosis via oxidative stress regardless of iron levels. This property makes copper complexes and copper chelators potential anticancer agents [47]. Here, we identify that copper chelator elesclomol is one such drug candidates by inducing ROS and ferroptosis in CRC. Although whether iron participates in this process remains unclear, the observed anticancer efficacy of elesclomol against CRC is significant and therefore deserves further investigation.

In this study, we also found that elesclomol‐induced ATP7A degradation is a prerequisite for ROS accumulation and ferroptosis in CRC cells. Intracellular copper transport is generally coordinated by ATP7A/B and CTR1/2, which is responsible for copper efflux and uptake, respectively. CTR1 is reported to be involved in the growth and drug resistance of CRC [48], but its protein level was not obviously changed in response to elesclomol treatment, suggesting the anti‐CRC effect of elesclomol may not be related to CTR1. Another copper transporter ATP7A is critical for many physiological processes, whereas dysregulation of ATP7A function has been associated with copper‐related metabolic disorders, including Menkes and Wilson diseases [49]. Moreover, ATP7A is also involved in tumorigenesis and cancer drug resistance. For example, the expression of ATP7A is higher in cancerous tissue compared with the adjacent tissue [50]. ATP7A is also overexpressed in cisplatin‐resistant nonsmall cell lung cancer (NSCLC) tissues compared with cisplatin‐sensitive NSCLC tissue [51]. Loss‐of‐function studies showed that silencing of ATP7A suppresses the growth and metastasis of breast and lung cancer [52]. These data suggest that ATP7A probably acts as an oncogene during cancer initiation and development. In agreement with these reports, we here found that elesclomol‐induced ATP7A degradation retards the proliferation of CRC cells, which is independent of copper chelation. In the present of copper supplementation, this ability to downregulate ATP7A leads to ferroptosis when copper is excessively enriched within cells, which endows elesclomol with robust anticancer efficiency superior to several other copper chelators. We also found that ATP7A protects SLC7A11 from degradation, whereas elesclomol‐mediated loss of ATP7A results in SLC7A11 downregulation, which further enhances oxidative stress to induce ferroptosis. These results indicate that ATP7A degradation is key for the antitumour activity of elesclomol. However, whether ATP7A is the direct target of elesclomol was not determined here. It has been reported that the mitochondrial reductase ferredoxin 1 (FDX1) is the direct target of elesclomol [53]. In brief, they found that elesclomol binds and inhibits FDX1, leading to copper‐dependent cell death in those proteotoxic stress‐resistant cancer cells [53]. This finding suggested elesclomol kills cancer cells via repressing FDX1‐mediated mitochondrial function, but hardly elucidated the mechanism of copper accumulation within mitochondria. Our study revealed that elesclomol degrades ATP7A thus blocking copper efflux, providing another layer of action mechanism of elesclomol. In addition, we also found that the expression of Cav‐1, a protein reported to regulate the stability of ATP7A, is not obviously changed upon treatment of elesclomol, suggesting that Cav‐1 may not be involved in elesclomol‐induced ATP7A degradation in CRC cells. Therefore, identification of E3 ligase for ATP7A will contribute to elucidate the underlying mechanism, which needs to be further explored.

## 5 Conclusions

In summary, our results demonstrate that elesclomol induces copper retention and subsequent ROS accumulation via degradation of ATP7A, leading to ferroptosis in CRC cells. This finding addresses the importance of ATP7A‐mediated copper metabolism in cancer cell growth and ferroptosis and suggests elesclomol as a potential therapeutic agent for CRC.

## Conflict of interest

The authors declare no conflict of interest.

## Author contributions

CH and JL conceived and designed the project. WG, ZH and JD performed the experiments. WG, ZH, JD and EN analysed the data. CH, JL and EN wrote the manuscript.

### Peer Review

The peer review history for this article is available at https://publons.com/publon/10.1002/1878‐0261.13079.

## Supporting information


**Fig. S1**. Elesclomol inhibits colorectal cancer cell growth.
**Fig. S2**. Elesclomol decreases the protein stability of ATP7A.
**Fig. S3**. Elesclomol downregulates the protein level of SLC7A11.
**Fig. S4**. Elesclomol decreases the protein level of SLC7A11 via ATP7A degradation.
**Fig. S5**. ATP7A degradation contributes to the antitumor effect of elesclomol.
**Table S1**. Primers for qPCR (5′ to 3′).

## Data Availability

The data that support the findings of this study are available from the corresponding author [hcanhua@scu.edu.cn] upon reasonable request.
