# Supplementary material for: Elesclomol induces copper‐dependent ferroptosis in colorectal cancer cells via degradation of ATP7A
Source: Mol Oncol. 2021 Sep 15;15(12):3527–44. doi: 10.1002/1878-0261.13079 (PMC8637554; doi:10.1002/1878-0261.13079)
Supplement: Supplementary file 1 — Fig. S1. Elesclomol inhibits colorectal cancer cell growth. Fig. S2. Elesclomol decreases the protein stability of ATP7A. Fig. S3. Elesclomol downregulates the protein level of SLC7A11. Fig. S4. Elesclomol decreases the protein level of SLC7A11 via ATP7A degradation. Fig. S5. ATP7A degradation contributes to the antitumor effect of elesclomol. Table S1. Primers for qPCR (5′ to 3′). [file MOL2-15-3527-s001.docx]

**Elesclomol induces copper-dependent ferroptosis in colorectal cancer cells via degradation of ATP7A**

Wei Gao^1#^, Zhao Huang^1#^, Jiufei Duan^1^, Edouard C. Nice^2^, Jie Lin^3^*, Canhua Huang^1^*

^1^ State Key Laboratory of Biotherapy and Cancer Center, West China Hospital, and West China School of Basic Medical Sciences and Forensic Medicine, Sichuan University, and Collaborative Innovation Center for Biotherapy, 610041, Chengdu, China

^2^ Department of Biochemistry and Molecular Biology, Monash University, Clayton, VIC, Australia

^3^ Department of Medical Oncology, The Second Affiliated Hospital of Kunming Medical University, Kunming, China.

^#^ These authors contribute equally to this work.

* Correspondence: [hcanhua@scu.edu.cn](mailto:hcanhua@scu.edu.cn); linjie@kmmu.edu.cn

**Supporting Information**

**Supporting figures**

**
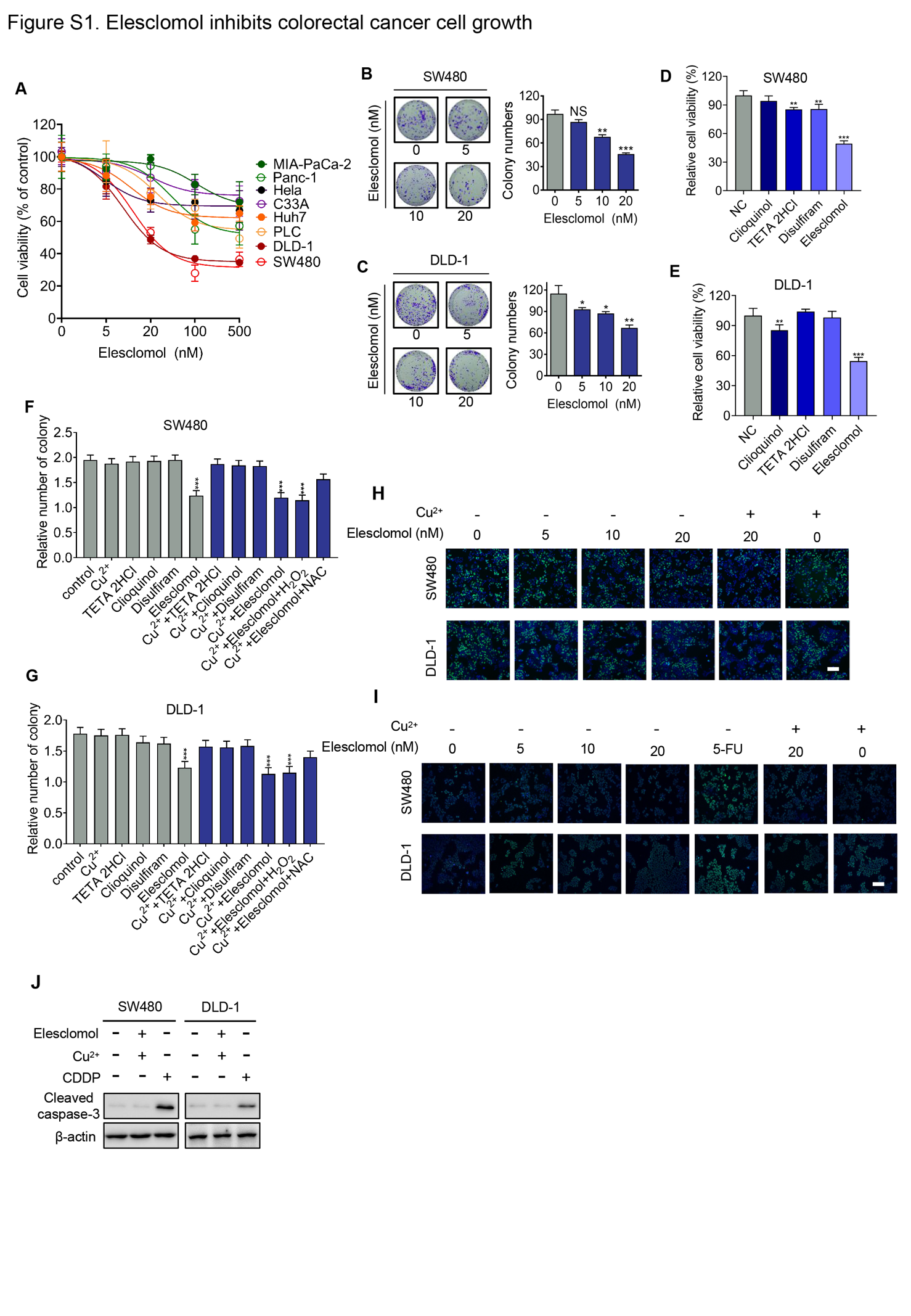
**

**Supporting Fig. S1. Elesclomol inhibits colorectal cancer cell growth**. (A) MTT assay showing viability of cells treated with indicated concentration of elesclomol for 24 hours (n=3). (B-C) Colony formation assay showing the cell proliferation of CRC cells treated with elesclomol at indicated concentration for two weeks (n=3). (D-E) MTT assay was performed to evaluate the antitumor effect of indicated copper chelators at a concentration of 20 nM for 24 hours (n=3). (F-G) Quantitation of survival fraction assay in Fig. 1D showing cell survival in the treatment of different copper chelators (n=3). (H) Representative images of EdU assay in Fig. 1E-F showing the proliferation of cells treated by elesclomol and copper. Scale bar, 50 μm. (I) Representative images of TUNEL assay in Fig. G-H showing cell apoptosis in the indicated treatment. Scale bar, 50 μm. (J) Western blot analysis showing the protein level of cleaved caspase-3 in cells treated with elesclomol and copper (n=3). Cisplatin (CDDP, 20 μM, 24 hours) was used as a positive control. Data are means ± SEM from at least 3 independent repeats. The P values were determined by two-tailed t-test. *P < 0.05, **P < 0.01; ***P < 0.001. NS, not significant.


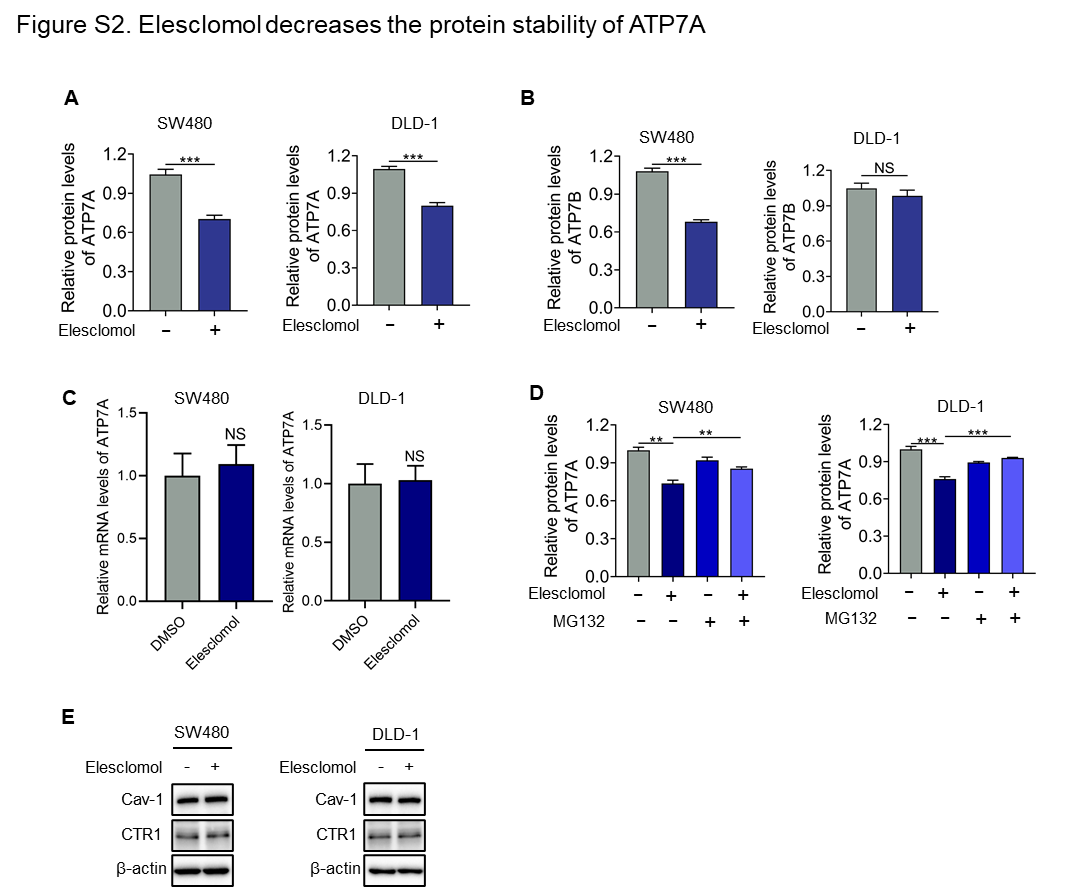


**Supporting Fig. S2.** **Elesclomol decreases the protein stability of ATP7A**. (A-B) Quantification of immunoblotting assay in Fig. 3B showing the protein levels of ATP7A and ATP7B in cells treated with 20 nM elesclomol for 24 hours (n=3). (C) qPCR assay showing the mRNA level of ATP7A in cells treated with 20 nM elesclomol for 24 hours (n=3). (D) Quantification of immunoblotting assay in Fig. 3M showing the protein stability of ATP7A in the treatment of elesclomol and MG132 (n=3). (E) Western blot showing the effect of elesclomol (20 nM, 24 hours) on the expression of indicated proteins (n=3). Data are means ± SEM from at least 3 independent repeats. The P values were determined by two-tailed t-test. **P < 0.01; ***P < 0.001. NS, not significant.

**
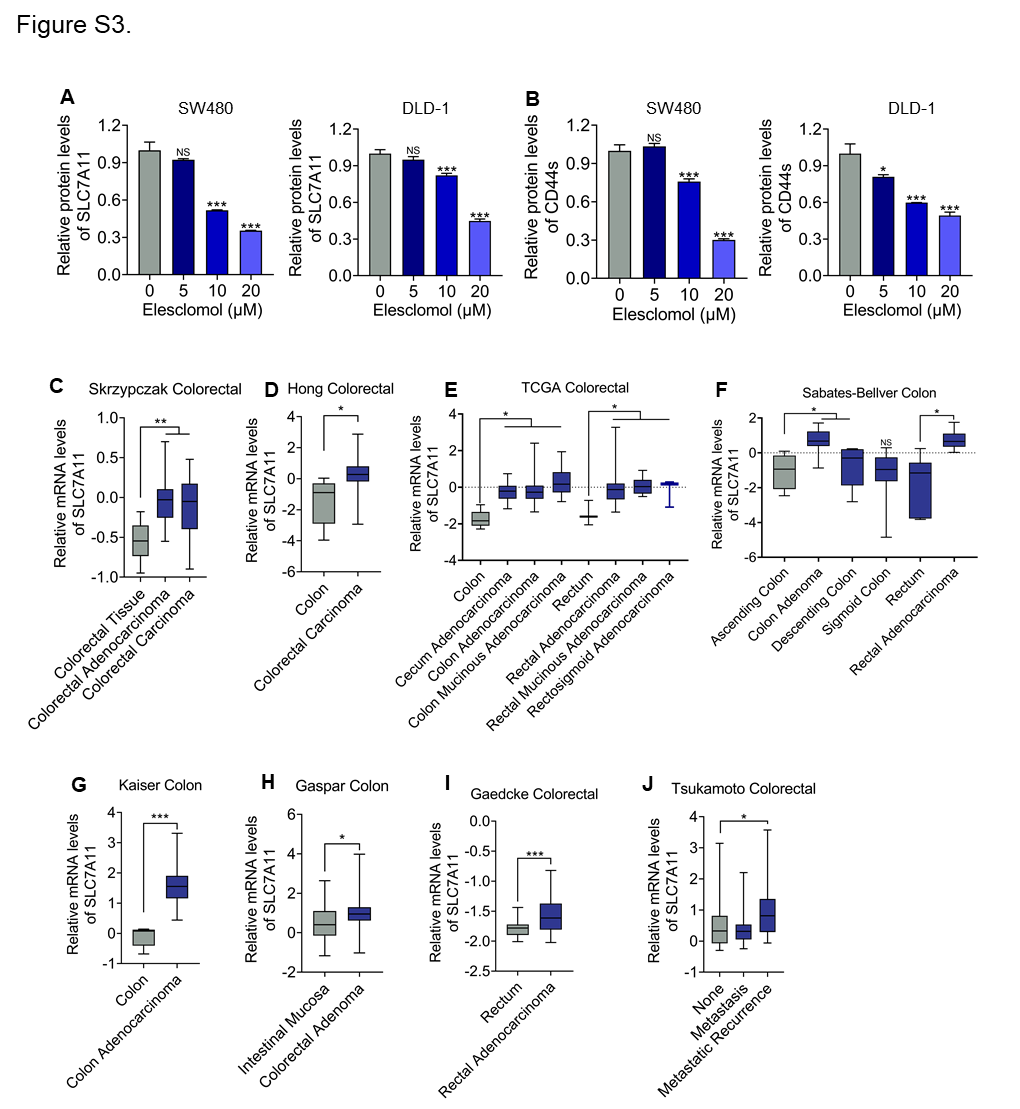
**

**Supporting Fig. S3.** **Elesclomol downregulates the protein level of SLC7A11**. (A-B) Quantification of immunoblotting assay in Fig. 4C showing the protein level of SLC7A11 and CD44 in cells treated with elesclomol at indicated concentration for 24 hours (n=3). (C-J) Dataset analysis showing the pathological significance of SLC7A11 in CRC clinic samples. Data are means ± SEM from at least 3 independent repeats. The P values were determined by two-tailed t-test. *P < 0.05, **P < 0.01; ***P < 0.001. NS, not significant.


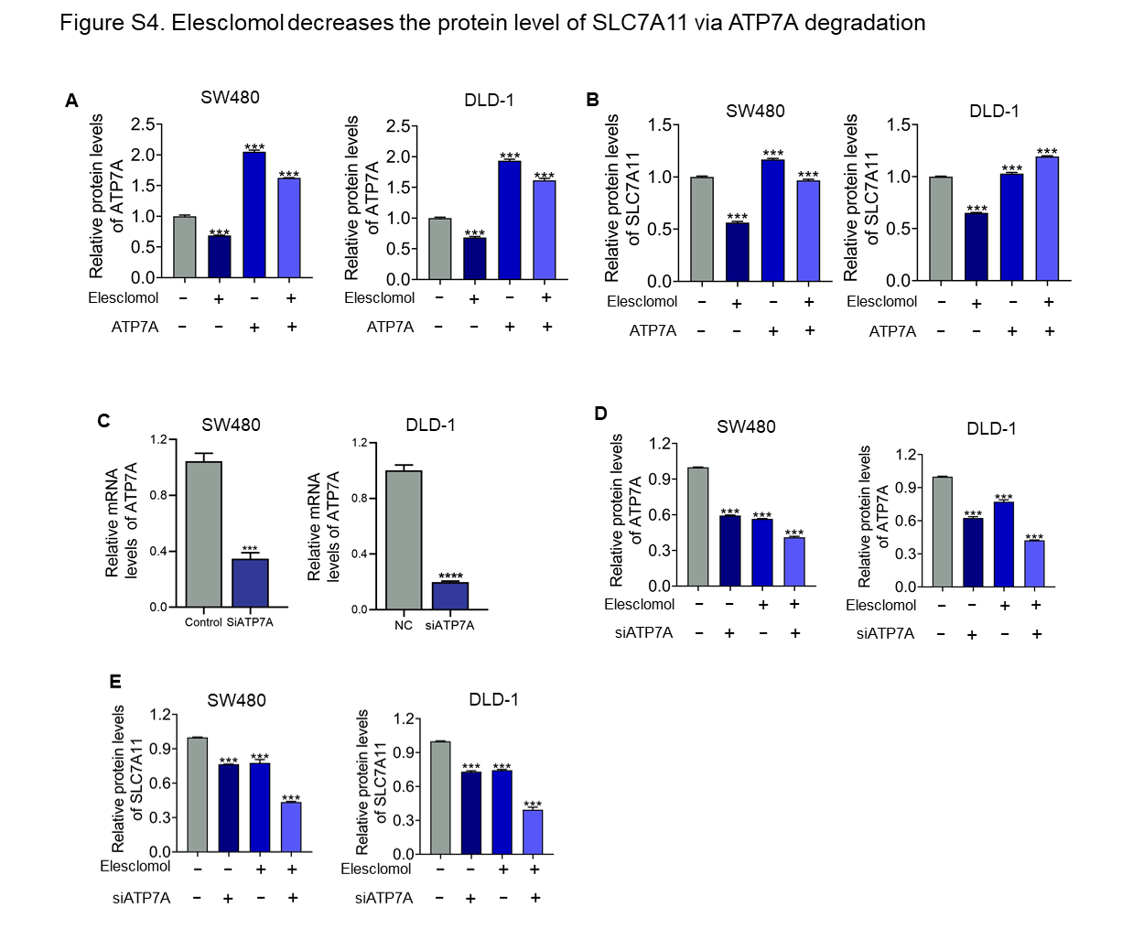


**Supporting Fig. S4. Elesclomol decreases the protein level of SLC7A11 via ATP7A degradation**. (A-B) Quantification of immunoblotting assay in Fig. 5A showing the protein level of ATP7A and SLC7A11 in cells (n=3). (C) qPCR assay showing mRNA level of ATP7A in cells treated with siRNA targeting endogenous ATP7A (n=3). (D-E) Quantification of immunoblot assay in Fig. 5B showing the protein level of ATP7A and SLC7A11 in cells (n=3). Data are means ± SEM from at least 3 independent repeats. The P values were determined by two-tailed t-test. ***P < 0.001.

**
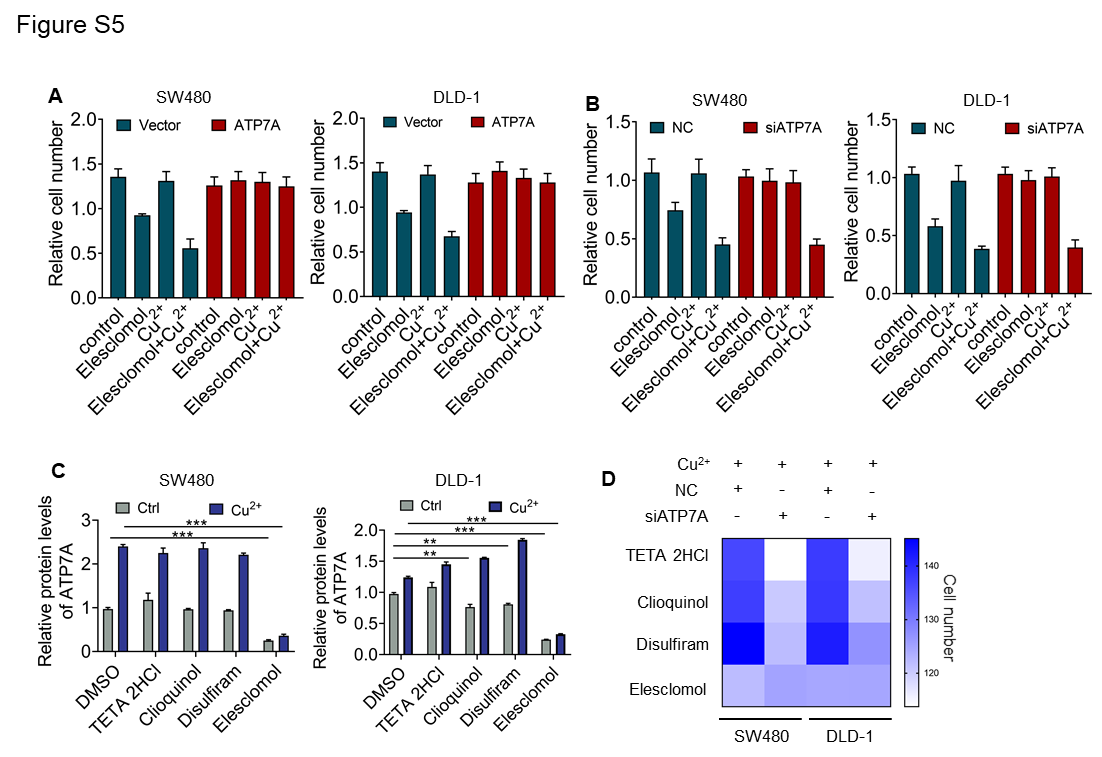
**

**Supporting Fig. S5.** **ATP7A degradation contributes to the antitumor effect of elesclomol**. (A-B) Quantification of survival fraction assay in Fig. 6A-B showing cell survival for the indicated treatment (n=3). (C) Quantification of immunoblotting assay in Fig. 6E showing the protein level of ATP7A in cells treated with indicated copper chelators (n=3). (D) Quantification of survival fraction assay in Fig. 6F showing the survival of cells in the treatment of indicated copper chelators with or without ATP7A knockdown (n=3). Data are means ± SEM from at least 3 independent repeats. The P values were determined by two-tailed t-test. **P < 0.01; ***P < 0.001.

**Table S1**

**Primers for qPCR (5’ to 3’)**

| SLC7A11-F | TCTCCAAAGGAGGTTACCTGC |
| --- | --- |
| SLC7A11-R | AGACTCCCCTCAGTAAAGTGAC |
| CAT-F | CGCAGAAAGCTGATGTCCTG |
| CAT-R | AAAGGCCCCTGCTCCTTTAG |
| SOD2-F | AAGGGAGATGTTACAGCCCAGATA |
| SOD2-R | TCCAGAAAATGCTATGATTGATATGAC |
| GPX1-F | CCAGTCGGTGTATGCCTTCT |
| GPX1-R | GATGTCAGGCTCGATGTCAA |
| SEPP1-F | CGTTGGAAGTGGTTGTGAC |
| SEPP1-R | CCATTGGAGTTTAGCATTGG |
| Txnrd1-F | GCCCTGCAAGACTCTCGAAATTA |
| Txnrd1-R | GCCCATAAGCATTCTCATAGACGA |
| ATP7A-F | TGTGTGCAGTCTATTGAGGGT |
| ATP7A-R | TGACAAGGTAGCATCAAATCCC |
